# Higher systemic immune-inflammation index is associated with increased risk of Parkinson’s disease in adults: a nationwide population-based study

**DOI:** 10.3389/fnagi.2025.1529197

**Published:** 2025-02-07

**Authors:** Jiayu Zhao, Zhipeng Wu, Fengyin Cai, Xuejv Yu, Zhenyu Song

**Affiliations:** ^1^Department of Neurology, Shandong First Medical University Affiliated Provincial Hospital, Jinan, Shandong, China; ^2^Department of Psychiatry, The Second Xiangya Hospital, Central South University, Changsha, Hunan, China; ^3^China National Clinical Research Center on Mental Disorders, Changsha, Hunan, China; ^4^Department of Nursing, The Fifth Affiliated Hospital of Guangzhou Medical University, Guangzhou, Guangdong, China

**Keywords:** systemic immune-inflammation index, Parkinson’s disease, inflammation, cross-sectional, risk factor

## Abstract

**Background:**

This study aimed to explore the association between a new inflammatory marker, systemic immune-inflammation index (SII), and the risk of Parkinson’s disease (PD) in adult population.

**Methods:**

A cross-sectional design was used, participants were recruited from the National Health and Nutrition Examination Survey (NHANES) from 2005 to 2020. Three logistic regression models were used to explore the association between SII and the risk of PD, and subgroup analysis and sensitivity analysis were used. In addition, the restricted cubic spline (RCS) was used to explore the dose-response relationship between SII and PD. Receiver operating characteristic (ROC) curves was used to explore the diagnostic value of SII for PD.

**Results:**

A total of 54,027 adults (mean age 35 years) were included in this study. The results of logistic regression showed that after adjusted for all covariates, compared with the Q1 group (lowest quartile in SII), the risk of PD in the Q3 group (OR = 1.82, 95%CI = 1.20–2.82, *p* < 0.001) and the Q4 group increased (OR = 2.49, 95%CI = 1.69–3.77, *p* < 0.001), with p-trend < 0.001. After excluding individuals with any missing values, sensitivity analysis also found a positive association between SII and PD. Subgroup analysis showed that this association was more significant in women, younger than 60 years old, non-smokers, alcohol drinkers, non-obese, and without a history of stroke, diabetes, or coronary heart disease. In addition, there was a positive dose-response relationship between SII and PD, and SII had an acceptable diagnostic value for PD (AUC = 0.72).

**Conclusion:**

SII is positively correlated with the prevalence of PD in the adult population, and SII can help differentiate between PD and non-PD cases.

## Introduction

Parkinson’s disease (PD) is a rapidly growing neurodegenerative disease (Dorsey et al., 2018). According to the latest international diagnostic criteria, PD is diagnosed by the presence of bradykinesia together with rigidity or tremor, along with supporting features (Bloem et al., 2021; Kobylecki, 2020). PD is considered the second most common neurodegenerative disease after Alzheimer’s disease (Ascherio and Schwarzschild, 2016). In developed countries, the median age-standardized annual incidence is 14 per 100,000 people in the total population and 160 per 100,000 people in people aged 65 years or older (Ascherio and Schwarzschild, 2016). It is generally believed that the age of onset of PD is mainly 60 years old (de Lau and Breteler, 2006), but several studies found that early-onset PD can appear before the age of 40, indicating that PD can occur at different ages (Gershanik, 2003; Riboldi et al., 2022; Schrag and Schott, 2006).

Among the exogenous factors known to influence the risk of Parkinson’s disease, such as exposure to pesticides (Brouwer et al., 2017), consumption of dairy products (Hughes et al., 2017), a history of melanoma (Gao et al., 2009), and traumatic brain injury (Lee et al., 2012), while a decreased risk is associated with smoking (Rose et al., 2024), caffeine consumption (Ross et al., 2000), elevated serum urate concentrations (Shen et al., 2013), physical activity (Fang et al., 2018), and use of ibuprofen (Gao et al., 2011) and other common medications (Ascherio and Schwarzschild, 2016), although these studies didn’t consider genetic factors (Trinh and Farrer, 2013), which is also associated with risk of PD. However, the pathogenesis of PD remains unclear. A large number of experimental and postmortem studies (Gate, 2022) have shown that inflammation plays an important role in the pathogenesis of PD. A study by Chen et al. (Chen et al., 2007) found that men with high plasma interleukin-6 concentrations had an increased risk of PD. Some case-control studies have found that IL-6, TNF-a, IL-1β, ST NFR 1, CRP, CCL 2, CX 3 CL 1, and CX CL 12 are elevated in the PD group (Qin et al., 2016; Qiu et al., 2019; Qu et al., 2023).

Systemic immune-inflammation index (SII) is a new type of systemic inflammation evaluation index that can objectively reflect the balance between host inflammatory and immune response status (Hu et al., 2014; Xu et al., 2024). An elevated SII usually suggests an elevated inflammatory status and weak immune response in patients (Hu et al., 2014). The calculation formula is the product of the platelet to neutrophil to lymphocyte ratio (Hu et al., 2014). SII has been found to be closely associated with the occurrence and prognosis of diseases in the elderly. A study by Tian et al. (2022) suggested that SII was associated with the prognosis of elderly patients with digestive system tumors. In terms of neurological diseases, Xu et al. (2024) conducted a study on 102 traumatic brain injuries (TBI) and found that the SII index increased in the early stages of TBI and was an independent risk factor for predicting poor prognosis in patients. Bao et al. (2023) found that SII was associated with cognitive impairment after acute ischemic cerebral infarction. Algul and Kaplan (2024) found that SII was associated with the severity of dementia in patients with Alzheimer’s disease. It is worth noting that SII was found to be negatively correlated with the motor performance of PD patients (Li et al., 2021). However, no study has yet explored the relationship between SII and the risk of PD in a large population sample.

To fill the gap of current studies, our study aims to explore the association between SII and the risk of PD in adults using data from a nationally representative cross-sectional survey, and further explore the diagnostic value of SII for the risk of PD. We hypothesize that individuals with higher SII are more likely to have a risk of PD and that SII can distinguish PD from non-PD in the population.

## Materials and methods

### Research design and research subjects

**FIGURE 1:**
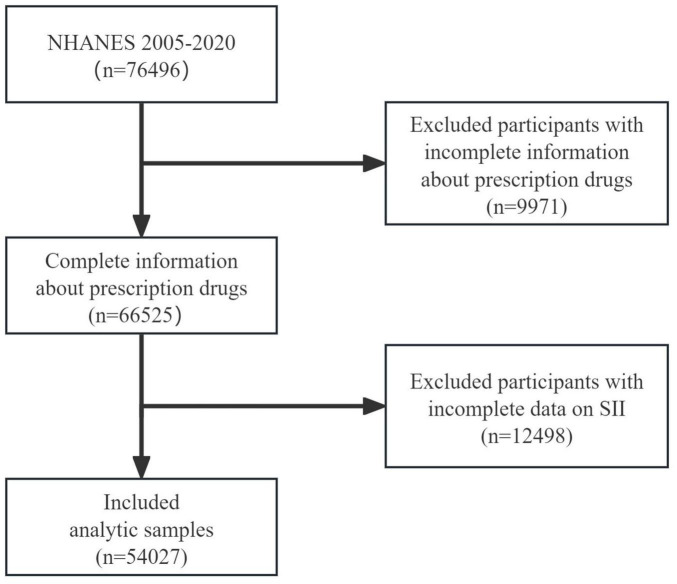
Flowchart of the participant selection from NHANES 2005–2020. NHANES, National Health and Nutrition Examination Survey; SII, systemic immune-inflammation index.

NHANES was constructed by the Centers for Disease Control and Prevention of the United States. The survey year is from 1999 to the present. The survey cycle is every 2 years. The sample size of each year is about 5000. The samples come from all over the country. NHANES includes demographic data, questionnaire data, dietary data, examination data and laboratory data. This study was approved by the National Cancer Institute and reviewed by the Health Statistics Research Ethics Review Committee. All participants signed informed consent. The research design and related data of NHANES can be downloaded at https://www.cdc.gov/nchs/nhanes/. The inclusion criteria of this study are as follows: (1) adults over 20 years old; (2) with prescription drug use data in the past 30 days; (3) with complete laboratory test data: platelet, neutrophil and lymphocyte data. After excluding all samples that did not meet the inclusion criteria, a total of 54,027 people were included in this study. The specific data flow chart is shown in Figure 1.

### Definition of PD

PD was defined based on the information provided by the subjects on prescription drug use in the past 30 days. Subjects were defined as having PD if they reported taking medication used to treat PD, including carbidopa, levodopa, methyldopa, benztropine, ropinirole, entacapone, and amantadine (Liu et al., 2023). With this definition, NHANES 2005-2020 reported 211 (1.06%) cases of PD in participants aged 45 and older, and 131 (1.27%) in participants aged 60 or older, whereas previous studies reported figures ranging from 0.47 to 0.77% of people with PD aged 45 and older in North America in 2012 (Willis et al., 2022), and 1% of people aged 65 or older with PD in industrialized countries in 2006 (de Lau and Breteler, 2006).

### Definition of systemic immune-inflammation index

Based on previous study (Hu et al., 2014), we calculated SII using the following formula:

$$S\,I\,I=\frac{P\,l\,a\,t\,e\,l\,e\,t\,c\,o\,u\,n\,t\times n\,e\,u\,t\,r\,o\,p\,h\,i\,l\,c\,o\,u\,n\,t}{l\,y\,m\,p\,h\,o\,c\,y\,t\,e\,c\,o\,u\,n\,t}$$

Platelet, neutrophil, and lymphocyte counts were derived from the CBC laboratory data of NHANES. The method used to derive CBC parameters was based on the Beckman Coulter counting and quantification method, combined with an automatic dilution and mixing device for sample processing, and a single-beam photometer for hemoglobin determination. WBC differentials used VCS technology (Volume, Conductivity, Scatter). For detailed detection procedures, please refer to the NHANES Laboratory/Medical Technician Procedure Manual (LPM) (CDC, 2020).

### Assessment of covariates

We included the following covariates: age (<60 years, ≥60 years), gender (male, female), race (Mexican American, other Hispanic, non-Hispanic white, non-Hispanic black, other race-including multi-racial), education (less than high school, high school or equivalent, college or above), marital status (married, widowed, divorced, separated, never married, living with partner), alcohol use (never, past drinker, current drinker), smoking (yes, no), BMI (<18.5, 18.5–24.9, 25.0–29.9, ≥ 30.0), sleep disorders (yes, no), hypertension (yes, no), diabetes (yes, no), coronary heart disease (yes, no), stroke (yes, no). Previous disease diagnosis information was based on self-reported information.

### Statistical methods

Since the NHANES database uses a complex, multi-stage sampling method for investigation, this study used 2-year MEC exam weights (WTMEC2YR)^1^ to perform weighted analysis of relevant indicators. First, we calculated the SII quartiles (first quartile = 296.47, median = 429.00, third quartile = 620.00) based on the SII levels of all participants and divided them into four groups according to these quartiles (see the distribution of SII levels in Supplementary Figure S1). Next, descriptive statistics were performed on the overall population and each group of participants. Continuous variables were described by mean and standard deviation, and categorical variables were described by frequency and percentage. The chi-square test was used to compare categorical variables between groups, and analysis of variance was used to compare continuous variables. Second, we used three logistic regression models to explore the relationship between SII levels and the risk of PD in the population. Model 1 (Crude model) did not adjust for covariates. Model 2 adjusted for age, gender, race, education level, marital status. Model 3 further adjusted for alcohol use, smoking-cigarette use, BMI, sleep disorders, hypertension, diabetes, coronary heart disease, and stroke based on Model 2. Next, we used subgroup analysis to explore the relationship between SII levels and the risk of PD in different populations. One model was fitted per covariate and adjusted for the other covariates, then we fit another adjusted model for each covariate with an interaction term between the covariate and the SII levels to study interaction effect. In addition, we excluded all individuals with missing values and performed sensitivity analysis to verify the robustness of our results. Finally, we used the restricted cubic spline (RCS) after adjusted for age, gender, race, education level, marital status, alcohol use, smoking-cigarette use, BMI, sleep disorders, hypertension, diabetes, coronary heart disease, and stroke to explore the dose-response relationship between SII levels and the risk of PD, we fit three models with three, four, five knots and the model with the lowest AIC was selected in our study (Frank and Harrell, 2015). Further, we used the Receiver operating characteristic (ROC) curves to detect the diagnostic value of SII on the risk of PD. All statistical analyses were performed using R (version 4.4.1). R package “rms” (version 6.9-0) was used to conduct RCS analysis. We used a two-sided test, *p* < 0.05 was considered statistically significant. For all models, the associations were reported with adjusted odds ratios (ORs), 95% confidence intervals (CIs), and *p*-values.

## Results

### General characteristics of the study population

A total of 54,027 people were included in this study, of which 49.5% were male, 50.5% were female, 78.7% were under 60 years old, 21.3% were 60 years old and above, the average age was 35 years old (SD, 24.1; Range, 20–85), 37.6% were non-Hispanic white, and 23.4% were non-Hispanic black. The number of patients diagnosed with PD was 260 (0.5%). We found that there were significant differences in the prevalence of PD among different SII level groups (*p* < 0.001). The distribution of SII was shown in Supplementary Figure S1. In addition, we found statistically significant group differences in age, race, marital status, BMI, alcohol consumption, sleep disorders, hypertension, diabetes, coronary heart disease, and stroke (all *p* < 0.001) (Table 1).

**TABLE 1:** Characteristics of study participants from NHANES 2005–2020^a^.

Variables	Total *n* = 54027	SII				*p*-value
		Q1(1.52–296.47) *n* = 13507	Q2(296.47–429.00) *n* = 13509	Q3(429.00–620.00) *n* = 13514	Q4(620.00–28397.27) *n* = 13497	
Weighted sample size	237,198,970	47,270,359	60,053,262	64,244,680	65,630,669	
Age (years), mean (SD)	35.08 (24.10)	27.50 (24.46)	34.23 (23.54)	37.79 (23.14)	40.82 (23.17)	< 0.001
Age distribution (years), n (%)						< 0.001
<60	42,493 (78.7)	11,333 (83.9)	1,0806 (80.0)	10,410 (77.0)	9,944 (73.7)	
≥ 60	11,534 (21.3)	2,174 (16.1)	2,703 (20.0)	3,104 (23.0)	3,553 (26.3)	
Gender (%)						0.509
Male	26,766 (49.5)	7,372 (54.6)	7,010 (51.9)	6,494 (48.1)	5,890 (43.6)	
Female	27,261 (50.5)	6,135 (45.4)	6,499 (48.1)	7,020 (51.9)	7,607 (56.4)	
Race, *n* (%)						< 0.001
Mexican American	10,070 (18.6)	2,190 (16.2)	2,559 (18.9)	2,687 (19.9)	2,634 (19.5)	
Other Hispanic	5,110 (9.5)	1,176 (8.7)	1,352 (10.0)	1,329 (9.8)	1,253 (9.3)	
Non-Hispanic White	20,293 (37.6)	3,806 (28.2)	4,920 (36.4)	5,489 (40.6)	6,078 (45.0)	
Non-Hispanic Black	12,662 (23.4)	4,773 (35.3)	3,083 (22.8)	2,555 (18.9)	2,251 (16.7)	
Other Race - Including Multi-Racial	5,892 (10.9)	1,562 (11.6)	1,595 (11.8)	1,454 (10.8)	1,281 (9.5)	
Education level, *n* (%)						0.353
Less than high school	8,362 (24.5)	1,547 (24.7)	2,072 (24.9)	2,258 (24.2)	2,485 (24.5)	
High school or equivalent	7,910 (23.2)	1,460 (23.3)	1,839 (22.1)	2,159 (23.1)	2,452 (24.2)	
College or above	17,793 (52.2)	3,267 (52.1)	4,405 (53.0)	4,926 (52.7)	5,195 (51.3)	
Marital status, *n* (%)						< 0.001
Married	18,279 (51.4)	3,303 (50.4)	4,623 (53.4)	5,058 (51.8)	5,295 (49.9)	
Widowed	4,029 (11.3)	734 (11.2)	890 (10.3)	1,100 (11.3)	1,305 (12.3)	
Divorced	4,341 (12.2)	816 (12.4)	1,024 (11.8)	1,168 (12.0)	1,333 (12.6)	
Separated	868 (2.4)	136 (2.1)	204 (2.4)	245 (2.5)	283 (2.7)	
Never married	6,021 (16.9)	1,186 (18.1)	1,401 (16.2)	1,616 (16.6)	1,818 (17.1)	
Living with partner	2,041 (5.7)	385 (5.9)	514 (5.9)	571 (5.9)	571 (5.4)	
Ratio of family income to poverty, n (%)						0.101
*≤* 1.00	13,101 (26.5)	3,609 (29.2)	3,180 (25.7)	3,182 (25.7)	3,130 (25.4)	
1.01–3.00	20,317 (41.1)	5,081 (41.1)	5,003 (40.4)	4,934 (39.9)	5,299 (43.0)	
> 3.00	16,014 (32.4)	3,680 (29.7)	4,190 (33.9)	4,243 (34.3)	3,901 (31.6)	
BMI (kg/m^2^), mean (SD)	26.24 (7.82)	23.61 (7.25)	25.76 (7.37)	27.13 (7.58)	28.30 (8.25)	< 0.001
BMI (kg/m^2^), *n* (%)						< 0.001
<18.5	8,884 (17.1)	3,870 (31.2)	2,315 (17.6)	1,520 (11.5)	1179 (9.0)	
18.5–24.9	15,575 (30.0)	3,768 (30.3)	4,120 (31.4)	4,030 (30.5)	3,657 (27.9)	
25.0–29.9	12,958 (25.0)	2,486 (20.0)	3,336 (25.4)	3,558 (27.0)	3,578 (27.3)	
≥ 30.0	14,429 (27.8)	2,295 (18.5)	3,362 (25.6)	4,084 (31.0)	4,688 (35.8)	
Alcohol use, *n* (%)						< 0.001
Never	7,798 (28.0)	1,482 (28.7)	1,891 (27.3)	2,021 (26.4)	2,404 (29.4)	
Past drinker	1,9575 (70.2)	3,598 (69.8)	4,892 (70.7)	5,490 (71.7)	5,595 (68.5)	
Current drinker	520 (1.9)	77 (1.5)	133 (1.9)	144 (1.9)	166 (2.0)	
Smoking—cigarette use, *n* (%)	7,047 (46.0)	1,242 (46.4)	1,571 (44.2)	1,945 (46.1)	2,289 (47.0)	0.586
Sleep disorders, *n* (%)	4,684 (12.3)	861 (12.0)	1,111 (11.8)	1,263 (12.1)	1,449 (12.9)	< 0.001
Hypertension, *n* (%)^b^	1,2475 (32.6)	2,246 (31.3)	2,856 (30.4)	3,389 (32.5)	3,389 (32.5)	< 0.001
Diabetes, *n* (%)^c^	4,462 (8.4)	768 (5.8)	1,072 (8.1)	1,146 (8.6)	1,476 (11.2)	< 0.001
Coronary heart disease, n (%)	1,394 (4.1)	241 (3.8)	307 (3.7)	386 (4.1)	460 (4.6)	< 0.001
Stroke, n (%)	1,407 (4.1)	238 (3.8)	273 (3.3)	362 (3.9)	534 (5.3)	< 0.001
Parkinson’s disease, n (%)	260 (0.5)	32 (0.2)	53 (0.4)	69 (0.5)	106 (0.8)	< 0.001

SII, systemic immune-inflammation index; BMI, body mass index; NHANES, National Health and Nutrition Examination Survey.

*^a^*All estimates accounted for sample weights and complex survey designs, and percentages and means were adjusted for survey weights of NHANES.

*^b^*Hypertension was defined based on self-reported information.

*^c^*Diabetes was defined as self-reported diabetes (participants who answered “yes” to the question “Has a doctor told you that you have diabetes?”).

### Associations between SII and PD

When SII was used as a numerical variable, without adjusting for any covariates, the higher the level of SII, the higher the risk of PD (OR = 1.02, 95%CI = 1.05–1.21, *p* = 0.003). After adjusting for age, gender, race, education level, and marital status, SII was positively correlated with the risk of PD (OR = 1.07, 95%CI = 1.02–1.14, *p* = 0.002). After further adjusting for alcohol use, smoking-cigarette use, BMI, sleep disorders, hypertension, diabetes, coronary heart disease and stroke, the relationship between SII and PD still held (OR = 1.07, 95%CI = 1.02–1.14, *p* = 0.006). When SII was treated as a categorical variable, in the three logistic regression models, compared with the SII level of first quartile (Q1), in model 1, the risk of PD in the Q2 group was significantly increased (OR = 1.66, 95%CI = 1.08–2.60, *p* = 0.024), which is also significant in Q3 (OR = 2.16, 95%CI = 1.43–3.33, *p* < 0.001) and Q4 group (OR = 3.33, 95% CI = 2.27–5.03, *p* < 0.001); In model 2, the risk of PD increased in group Q3 (OR = 1.67, 95%CI = 1.10–2.58, *p* = 0.018) and Q4 (OR = 2.29, 95%CI = 1.55–3.47, *p* < 0.001). In model 3, the risk of PD in group Q3 (OR = 1.82, 95%CI = 1.20–2.82, *p* < 0.001) and group Q4 (OR = 2.49, 95%CI = 1.69–3.77, *p* < 0.001) was increased, and the three models showed significant trends of increasing risk of PD with increasing SII levels (all *p*-trend < 0.001) (Table 2). Detailed summaries of multivariate logistic regressions were shown in Supplementary Table S2.

**TABLE 2:** Associations between SII levels and the risks of Parkinson’s diseases^a^.

SII	Model1			Model 2			Model 3		
	**OR**	**95% CI**	**p-value**	**OR**	**95% CI**	***p*-value**	**OR**	**95% CI**	***p*-value**
As continuous (per SD)	1.12	(1.05, 1.21)	0.003	1.07	(1.02,1.14)	0.002	1.07	(1.02,1.14)	0.006
InterquartileQuartile 1(1.52–296.47)	Ref.			Ref.			Ref.		
Quartile 2(296.47–429.00)	1.66	(1.08, 2.60)	0.024	1.42	(0.92, 2.23)	0.117	1.53	(0.98, 2.40)	0.059
Quartile 3(429.00–620.00)	2.16	(1.43, 3.33)	< 0.001	1.67	(1.10, 2.58)	0.018	1.82	(1.20, 2.82)	< 0.001
Quartile 4(620.00–28397.27)	3.33	(2.27, 5.03)	< 0.001	2.29	(1.55, 3.47)	< 0.001	2.49	(1.69, 3.77)	< 0.001
p-trend	< 0.001			<0.001			< 0.001		

SII, systemic immune-inflammation index; NHANES, National Health and Nutrition Examination Survey. OR, odds ratio; CI, confidence interval. ^a^ The associations between SII levels and the risks of Parkinson’s disease are presented as ORs (95% CI). Model 1 did not adjust for any covariates. Model 2 adjusted for age (years), gender, race, education level, marital status. Model 3 further adjusted for alcohol use, smoking—cigarette use, body mass index, sleep disorders, hypertension, diabetes, coronary heart disease and stroke based on Model 2.

### Subgroup analysis and sensitivity analysis

The results of subgroup analysis showed that, compared with Q1, the association between high level SII (Q4) and increased risk of PD was more likely to be found in female (OR = 3.40, 95%CI = 1.91–6.50, *p* < 0.001), younger than 60 years old (OR = 3.67, 95%CI = 1.99–7.31, *p* < 0.001), non-Hispanic white (OR = 3.04, 95%CI = 1.71–5.90, *p* < 0.001), high school education or above (OR = 5.98, 95%CI = 2.89–14.5, *p* < 0.001), not currently smoking (OR = 3.94, 95%CI = 2.20–7.70, *p* < 0.001), alcohol drinker (OR = 3.20, 95%CI = 1.86–5.87, *p* < 0.001), non-obese (OR = 3.38, 95%CI = 1.97–6.12, *p* < 0.001), without a history of stroke (OR = 2.56, 95%CI = 1.70–3.96, *p* < 0.001), without a history of diabetes (OR = 3.31, 95% CI = 2.08–5.51, *p* < 0.001), and without a history of coronary heart disease (OR = 2.63 95%CI = 1.75–4.07, *p* < 0.001) (Table 3). We conducted a sensitivity analysis for missing values. After excluding subjects with missing values in any covariate, compared with the low-level SII group, the high-level SII group still had a higher risk of PD (Table 4).

**TABLE 3:** Subgroup analysis of associations between SII levels and the risks of Parkinson’s diseases^a^.

	ORs (95% CI)				p for interaction
**Variable**	**Q1**	**Q2**	**Q3**	**Q4**	
**Gender**					
Male	Ref.	1.28 (0.70, 12.37)	1.66 (0.94, 3.00)	2.08 (1.22, 3.68)*	0.026
Female		1.93 (1.01, 3.87)*	2.25 (1.22, 4.44)*	3.40 (1.91, 6.50)^***^	
**Age (years)**					
< 60	Ref.	1.98 (1.01, 4.12)*	2.38 (1.24, 4.87)*	3.67 (1.99, 7.31)^***^	0.001
≥ 60		1.20 (0.67, 2.15)	1.43 (0.84, 2.50)	1.78 (1.09, 3.05)*	
Race					0.034
Non-Hispanic White	Ref.	1.82 (0.95, 3.70)	1.91 (1.03, 3.82)	3.04 (1.71, 5.90)^***^	
Other race		1.20 (0.64, 2.22)	1.58 (0.88, 2.86)	1.20 (0.64, 2.24)	
**Education level**					
High school and below	Ref.	0.85 (0.48, 1.50)	1.01 (0.59, 1.14)	1.49 (0.92, 2.47)	0.076
Above high school		3.98 (1.84, 9.91)^**^	4.72 (2.23, 11.6)^***^	5.98 (2.89, 14.5)^***^	
**Smoking—cigarette use**					
Every day or some day	Ref.	1.14 (0.61, 2.11)	1.30 (0.73, 2.35)	1.54 (0.90, 2.71)	0.161
Not at all		2.19 (1.15, 4.45)*	2.67 (1.44, 5.33)^**^	3.94 (2.20, 7.70)^***^	
**Alcohol use**					
Yes	Ref.	1.75 (0.94, 3.35)	2.14 (1.20, 4.03)*	3.20 (1.86, 5.87)^***^	0.001
No		1.33 (0.71, 2.53)	1.53 (0.84, 2.86)	1.88 (1.08, 3.41)*	
**BMI (kg/m^2^)**					
Normal (< 25)	Ref.	1.80 (0.97, 3.43)	2.42 (1.37, 4.49)^**^	3.38 (1.97, 6.12)^***^	0.519
Overweight/Obese (≥25)		1.19 (0.64, 2.27)	1.20 (0.66, 2.25)	1.55 (0.89, 2.83)	
**Hypertension**					
Yes	Ref.	1.45 (0.83, 2.59)	1.40 (0.81, 2.46)	2.08 (1.28, 3.55)^**^	< 0.001
No		1.61 (0.79, 3.40)	2.52 (1.32, 5.12)^**^	3.14 (1.69, 6.27)^***^	
**Diabetes**					
Yes	Ref.	0.81 (0.35, 1.89)	0.98 (0.45, 2.21)	0.94 (0.46, 2.60)	0.486
No		1.87 (1.12, 3.24)*	2.21 (1.35, 3.75)^**^	3.31 (2.08, 5.51)^***^	
**Stroke**					
Yes	Ref.	1.77 (0.42, 8.76)	2.43 (0.72, 1.10)	2.05 (0.63, 9.15)	0.763
No		1.51 (0.95, 2.42)	1.74 (1.12, 2.77)*	2.56 (1.70, 3.96)^***^	
**Coronary heart disease**					
Yes	Ref.	1.87 (0.49, 8.95)	0.92 (0.21, 4.60)	1.39 (0.39, 6.42)	0.728
No		1.49 (0.94, 2.41)	1.93 (1.25, 3.05)^**^	2.63 (1.75, 4.07)^***^	

SII, systemic immune-inflammation index; OR, odds ratio; CI, confidence interval.

*^a^*The associations between SII levels and the risks of Parkinson’s disease are presented as ORs (95% CI). Model adjusted for age (years), gender, race, education level, marital status, alcohol use, smoking—cigarette use, body mass index, sleep disorders, hypertension, diabetes, coronary heart disease and stroke. *p-value < 0.05, **p-value < 0.01, and ***p-value < 0.001.

**TABLE 4:** Associations between SII levels and the risks of Parkinson’s diseases among participants without missing values^a^.

SII	Model1			Model 2			Model 3		
	**OR**	**95% CI**	***p*-value**	**OR**	**95% CI**	***p*-value**	**OR**	**95% CI**	***p*-value**
As continuous (per SD)	1.01	(1.01,1.01)	0.001	1.01	(1.01,1.01)	0.011	1.01	(1.01,1.01)	0.011
InterquartileQuartile 1(1.52—296.47)	Ref.			Ref.			Ref.		
Quartile 2(296.47—429.00)	1.33	(0.56, 3.27)	0.513	1.19	(0.50, 2.94)	0.689	1.35	(0.56, 3.32)	0.500
Quartile 3(429.00–620.00)	1.34	(0.56, 3.28)	0.513	1.13	(0.47, 2.79)	0.785	1.34	(0.56, 3.31)	0.505
Quartile 4(620.00–28397.27)	2.91	(1.41, 6.58)	0.005	2.35	(1.13, 5.36)	0.029	2.70	(1.30, 6.13)	0.011
p-trend			0.002			0.018			0.006

SII, systemic immune-inflammation index; OR, odds ratio; CI, confidence interval.

*^a^*The associations between SII levels and the risks of Parkinson’s disease are presented as ORs (95% CI). Model 1 did not adjust for any covariates. Model 2 adjusted for age (years), gender, race, education level, marital status. Model 3 further adjusted for alcohol use, smoking—cigarette use, body mass index, sleep disorders, hypertension, diabetes, coronary heart disease, and stroke based on Model 2.

### Dose-response relationship between SII and PD

**FIGURE 2:**
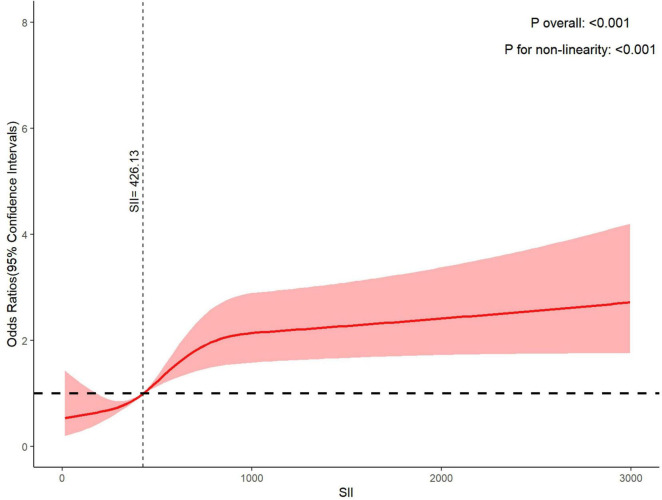
Dose-response relationship between systemic immune-inflammation index and Parkinson’s disease.

Four knots were selected in our study, details of model fit were shown in Supplementary Table S3. The RCS curve results showed that after adjusting for age, gender, race, education level, marital status, alcohol drinking, smoking-cigarette use, BMI, sleep disorders, hypertension, diabetes, coronary heart disease and stroke, the higher the level of SII, the higher the risk of PD, a non-linear relationship was found (p overall < 0.001, p for non-linearity < 0.001), and SII > 426.13 indicated a steep increased risk of PD (Figure 2).

### The diagnostic value of SII for PD

**FIGURE 3:**
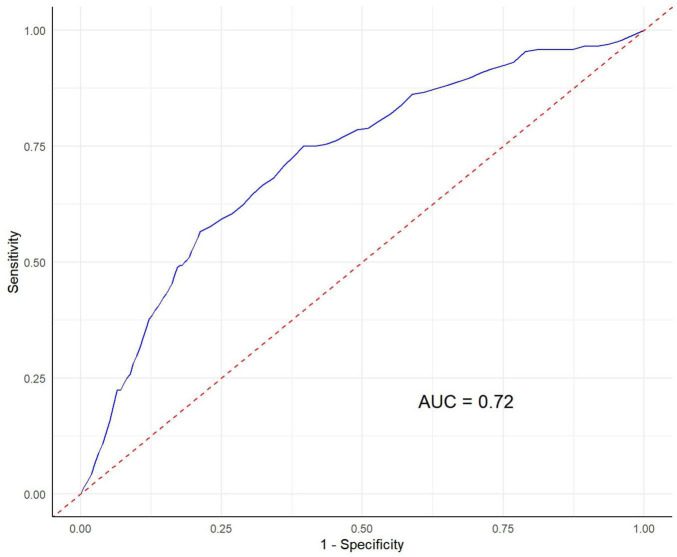
The diagnostic value of systemic immune-inflammation index for Parkinson’s disease risk. AUC, Area Under the Curve.

The ROC curve results showed that the level of SII can distinguish PD from non-PD in the population (AUC = 0.72, 95% CI: 0.69–0.75) (Figure 3). In addition, the accuracy of the model was 0.76, the sensitivity was 0.79, the specificity was 0.71, and Youden index was 0.50.

## Discussion

Our study is the first to examine the relationship between SII levels and risk of PD in a large, nationally representative cross-sectional adult population sample. We found that the higher the SII level, the higher the risk of adult PD, and this trend is very significant, especially among women, younger than 60 years old, non-Hispanic white, high school education or above, not currently smoking, alcohol drinker, non-obese, without a history of stroke, diabetes, or coronary heart disease. In addition, we found a positive dose-response relationship between SII levels and the risk of PD, and SII levels can distinguish PD from non-PD in adult population.

The relationship between SII and the risk of PD has not been studied, but a large number of previous studies have shown that peripheral blood inflammatory markers play an important role in the occurrence and development of PD. The results of a case-control study on early PD by Kim et al. (2018) showed that the levels of IL-1β, IL-2 and IL-6 in the PD group were significantly higher than those in the control group, and IL-10 was associated with patients’ non-motor symptoms. Studies have shown that chronic proinflammatory states already exist in the prodromal stage of PD, such as iRBD (Lindestam Arlehamn et al., 2020; Stokholm et al., 2017; Sulzer et al., 2017). A study by Chen et al. (2007) showed that men with high plasma IL-6 levels have an increased risk of PD, but the sample size was small and there was a lack of discussion of other inflammatory biomarkers. A meta-analysis showed that PD patients had higher levels of IL-6, tumor necrosis factor-α, IL-1β, IL-2, IL-10, C-reactive protein and RANTES in peripheral blood, which further strengthened the clinical evidence that PD patients have peripheral inflammatory responses (Qin et al., 2016). Moreover, a case-control study by Pedersen et al. (2023) found nine CSF inflammatory markers associated with PD (increased levels of CD5, CDCP1, IL-18R1, and IL-6 and decreased levels of ADA, CCL23, CD8A, FGF-19, and MCP-2).

On the basis of the above studies, our study further explored the relationship between SII, a new systemic inflammatory response index based on peripheral blood inflammatory cells, and the risk of PD. The calculation of SII is based on the counts of platelets, neutrophils and lymphocytes, that is, the product of platelets and the ratio of neutrophils and lymphocytes (Hu et al., 2014). These peripheral blood inflammatory markers are associated with the risk of PD. Platelets originate from megakaryocytes in the bone marrow and are defined as very small anucleated cell fragments with a diameter of 2-4 μm, which are involved in thrombosis (Beura et al., 2022). Platelet dysfunction is believed to be related to endothelial cell damage, and platelets can express neuron-specific molecules and receptors; it also expresses several PD-specific biomarkers, such as α-synuclein, parkin, PTEN-induced kinase 1, tyrosine hydroxylase, and dopamine transporter (Beura et al., 2022). Therefore, platelets are often used to build peripheral models of PD, and antiplatelet drugs are considered to have potential therapeutic value for PD.

Neutrophils are immune cells with unique biological characteristics and strong antimicrobial properties (Burn et al., 2021). These cells phagocytose and subsequently kill prokaryotes and eukaryotes very effectively. The neutrophil-to-lymphocyte ratio (NLR) is a complete blood count (CBC)-based biomarker that reflects the balance between immunity and systemic inflammation (Hosseini et al., 2023). Studies have found that NLR in the plasma of PD patients is significantly higher than that of HC (Li et al., 2024; Munoz-Delgado et al., 2021). The results also highlighted the correlation between plasma NLR and the total UPDRS score and UPDRS I-III score in PD patients (Li et al., 2024), indicating that NLR is associated with the severity of symptoms in PD patients. Neutrophils are able to penetrate the epithelial and vascular wall cell layers and promote the body’s inflammatory response by regulating chemokines (Ley et al., 2018). An animal experiment by Jensen et al. (2021) found that T cell infiltration was detected in the hippocampus, neocortex, striatal perivascular area, and parenchyma of PD mice, and lower lymphocyte counts were associated with a greater risk of PD. In addition, the study by Dommershuijsen et al. (2022) found that the higher the lymphocyte count, the lower the likelihood of developing PD. Some studies have shown that NLR is higher in PD patients (Munoz-Delgado et al., 2021).

In addition, this study also found that the relationship between SII and the risk of PD has certain heterogeneity in different subgroups of people, which may be related to the differences in the risk of PD in different groups. Gender is considered an important risk factor for PD, with men having a higher incidence of PD than women, and a large number of studies have shown that there are also differences in symptoms, development, and response to treatment between male and female PD patients (Collaborators, 2019; Hirsch et al., 2016). Although sex differences in the association of SII with PD have not been studied, one longitudinal study showed (Cheng et al., 2024) that the impact of SII on mortality in male and female hypertensive patients was significantly different, with women with higher SII levels having a higher risk of death. Similar to the findings of this study, SII is more significantly associated with poor prognosis in women. Wei et al. (2024) found that in an age-stratified analysis, increased SII was a better predictor of the risk of metabolic syndrome in young people than in older people. This shows that SII can better reflect the systemic inflammatory status of young and middle-aged people. Inflammatory factors in young people are precisely and strictly regulated and are at lower levels (Singh and Newman, 2011). Elderly people are often in a state of chronic inflammation (De Maeyer et al., 2020; Sarkar and Fisher, 2006). Therefore, fluctuations in SII levels in young people are more sensitive to disease. Similarly, previous studies have shown (Demir and Demir, 2023) that compared with non-smokers, smokers have higher SII levels and are in a chronic inflammatory state for a long time. Therefore, the link between changes in SII levels and PD risk is more obvious among non-smokers. In terms of alcohol use, people who drink a small or moderate amount of beer have a lower risk of PD, while people who drink a higher amount of wine have an increased risk of PD (Imhof et al., 2001). It was found that moderate drinking can reduce the body’s inflammation level (Zhang et al., 2014). Our results suggest that among people who drink alcohol, the link between SII and PD risk is more significant. Further research is needed to identify this mechanism.

Our study found that the relationship between SII and the occurrence of PD in non-obese people is more significant, which may be related to the fact that the disease onset of non-obese people is more sensitive to fluctuations in SII levels. Zhou et al. (2024) found that obese people have higher SII levels, suggesting that inflammation may play an important role in the occurrence and development of obesity, and obesity may also lead to a sustained inflammatory response in the body. Nie et al. (2023) found that elevated SII has a stable relationship with an increased risk of diabetes, indicating that patients with diabetes have higher levels of inflammation. A prospective cohort study involving 13,929 adults by Xu et al. (2021) found that high SII was associated with increased risk of total stroke and ischemic stroke. A large number of previous studies have found that SII has a certain predictive effect on the occurrence of coronary heart disease (Yang et al., 2020) and is associated with the severity of coronary heart disease (Candemir et al., 2021). Our results concluded that SII levels are only associated with the risk of PD in people without coronary heart disease, stroke, or diabetes. On the one hand, the link between SII and PD risk is more significant in people with lower inflammation levels. This may be related to the fact that changes in the body’s SII levels are more sensitive to indicating the occurrence of PD in a low-inflammatory state. The specific mechanism needs to be further studied. On the other hand, pharmacological interventions and primary prevention measures targeting these diseases may attenuate the association between SII and PD. Insulin resistance is closely related to chronic inflammation, and the use of antidiabetic drugs may improve insulin resistance and reduce chronic inflammation (Shoelson et al., 2006). Medications such as statins and aspirin help reduce inflammation in people with coronary artery disease (Cheng et al., 2022; Shoelson et al., 2006).

Another important finding is that our results suggested a clear dose-response relationship between SII and the increased risk of PD, and high levels of SII can differentiate the occurrence of PD in the population with acceptable performance. Although the diagnostic value of SII for the risk of PD in the population has not been explored, previous studies have explored the predictive value of SII for other neurological diseases. Cheng et al. (2023) found that SII and SIRI can better predict the occurrence of stroke in 5907 asthma patients, and SIRI has a better predictive value for stroke prevalence than SII. Algul et al. found that SII, as a new inflammatory marker, is related to the severity of dementia in AD patients (Algul and Kaplan, 2024). In the study of the relationship between other inflammatory markers and PD, the neutrophil-to-lymphocyte ratio (NLR), lymphocyte-to-monocyte ratio (LMR) and neutrophil-to-high-density lipoprotein ratio (NHR) are considered to be able to predict the occurrence of PD, among which NLR has a relatively better predictive value (Li et al., 2024). The findings of this study are an important supplement to the association of PD by inflammatory markers. Whether the diagnostic value of SII for PD is different from that of other inflammatory markers needs further exploration.

## Limitation

Although this study has some important findings related to PD, we have several limitations. First, we used a cross-sectional design and cannot directly infer causality. Thus, the association between SII and PD needs to be further explored in longitudinal studies. Second, the assessment of PD is based on self-reported prescription medication data, and some subjects may have recall bias. Consequently, patients with non-PD parkinsonism may have been inadvertently included. Finally, participants of our study are from American population, and the results cannot be applied to the world population. This finding needs to be verified in populations from different countries.

## Conclusion

In summary, our study is the first to find an association between high levels of SII and the risk of PD in a nationally representative cross-sectional sample, and further explore the heterogeneity of this association in different populations. In addition, we also found a positive dose-response relationship between SII and PD, and SII level has a good diagnostic value for the risk of PD in the population. Since SII is a new, valuable, and easy-to-measure inflammatory marker, this study provides important insights into the exploration of risk factors for PD and its prevention.

## Data Availability

The datasets presented in this study can be found in online repositories. The names of the repository/repositories and accession number(s) can be found below: The related data of NHANES can be downloaded at https://www.cdc.gov/nchs/nhanes/.

## Footnotes

1
https://wwwn.cdc.gov/nchs/nhanes/tutorials/weighting.aspx
