# Supplementary material for: Higher systemic immune-inflammation index is associated with increased risk of Parkinson’s disease in adults: a nationwide population-based study
Source: Front Aging Neurosci. 2025 Feb 7;17:1529197. doi: 10.3389/fnagi.2025.1529197 (PMC11842390; doi:10.3389/fnagi.2025.1529197)
Supplement: Supplementary file 1 [file Table_1.doc]

| Supplementary Table 1. Characteristics of PD patients identified in our study. | |
| --- | --- |
| Variables | N=260 |
|
| Weighted sample size | 1245223 |
| Medication duration (years), mean (SD) | 5.9 (7.2) |
| Number of medication, mean (SD) | 7.3 (4.1) |
| Age (years), mean (SD) | 35.08 (24.10) |
| Age distribution (years), n (%) |  |
| <60 | 113 (43.5) |
| ≥60 | 147 (56.5) |
| Gender(%) |  |
| Male | 123 (47.3) |
| Female | 137 (52.7) |
| Race, n (%) |  |
| Mexican American | 26 (10.0) |
| Other Hispanic | 16 (6.2) |
| Non-Hispanic White | 168 (64.6) |
| Non-Hispanic Black | 39 (15.0) |
| Other Race - Including Multi-Racial | 11 (4.2) |
| Education level, n (%) |  |
| High school and below | 129 (51.4) |
| Above high school | 122 ( 48.6) |
| Marital status, n (%) |  |
| Married | 124 (49.0) |
| Widowed | 58 (22.9) |
| Divorced | 31 (12.3) |
| Separated | 2 (0.8) |
| Never married | 31 (12.3) |
| Living with partner | 7 (2.8) |
| Ratio of family income to poverty, n (%) |  |
| *≤*1.00 | 55 (23.1) |
| 1.01–3.00 | 114 (47.9) |
| >3.00 | 69 (29.0) |
| Body mass index (kg/m2 ), mean (SD) | 30.63 (7.40) |
| Body mass index (kg/m2 ), n (%) |  |
| <18.5 | 5 (2.0) |
| 18.5–24.9 | 57 (23.4) |
| 25.0–29.9 | 66 (27.0) |
| *≥*30.0 | 116 (47.5) |
| Alcohol use, n (%) |  |
| Never | 69 (43.4) |
| Past drinker | 90 (56.6) |
| Current drinker | 0 (0.0) |
| Smoking—cigarette use, n (%) | 54 (43.2) |
| Sleep disorders, n (%) | 84 (33.7) |
| Hypertension, n (%) | 152 (60.3) |
| Diabetes, n (%) | 57 (22.7) |
| Coronary heart disease, n (%) | 24 (9.7) |
| Stroke, n (%) | 30 (12.0) |


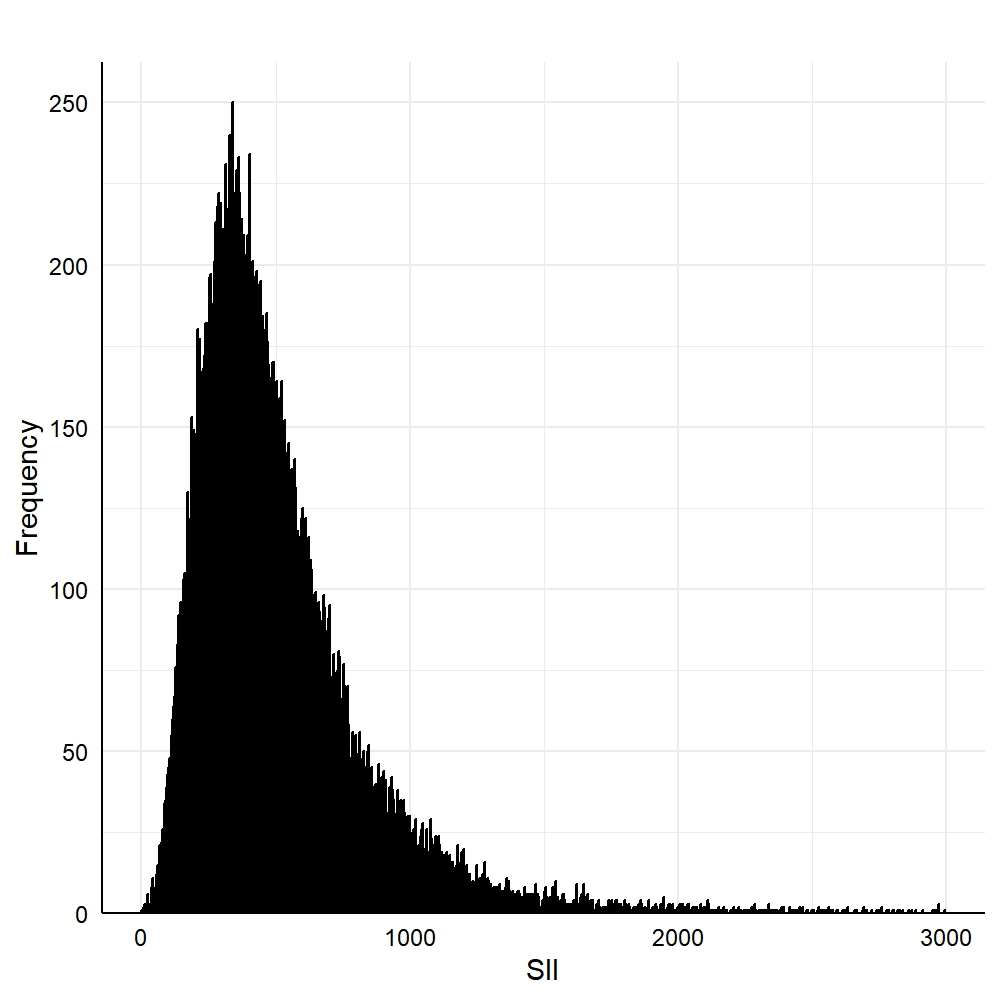


Supplementary Figure S1. Distribution of systemic immune-inflammation index.

| Supplementary Table 2. Complete summary of multivariate logistic regressions^a^. | | | | | | | | |
| --- | --- | --- | --- | --- | --- | --- | --- | --- |
|  | OR | B | SE | z value | 95%CI | | AIC | p-value |
|  |  |  |  |  | Lower | Upper |  |  |
| **Model 1** |  |  |  |  |  |  |  |  |
| As continuous (per SD) | 1.12 | 0.11 | 0.03 | 2.89 | 1.05 | 1.21 | 3283.4 | 0.003 |
| Interquartile |  |  |  |  |  |  |  |  |
| Quartile1 (1.52–296.47) | Ref. |  |  |  |  |  |  |  |
| Quartile2 (296.47–429.00) | 1.66 | 0.50 | 0.22 | 2.26 | 1.08 | 2.60 | 3256.5 | 0.024 |
| Quartile3 (429.00–620.00) | 2.16 | 0.77 | 0.21 | 3.60 | 1.43 | 3.33 |  | <0.001 |
| Quartile4 (620.00–28397.27) | 3.33 | 1.20 | 0.2 | 5.96 | 2.27 | 5.03 |  | <0.001 |
| p-trend |  |  |  |  |  |  |  |  |
| **Model 2** |  |  |  |  |  |  |  |  |
| As continuous (per SD) | 1.07 | 0.07 | 0.02 | 3.01 | 1.02 | 1.14 | 3093.2 | 0.002 |
| Interquartile |  |  |  |  |  |  |  |  |
| Quartile1 (1.52–296.47) | Ref. |  |  |  |  |  |  |  |
| Quartile2 (296.47–429.00) | 1.42 | 0.35 | 0.22 | 1.57 | 0.92 | 2.23 | 3084.1 | 0.117 |
| Quartile3 (429.00–620.00) | 1.67 | 0.51 | 0.21 | 2.36 | 1.10 | 2.58 |  | 0.018 |
| Quartile4 (620.00–28397.27) | 2.29 | 0.82 | 0.2 | 4.03 | 1.55 | 3.47 |  | <0.001 |
| p-trend |  |  |  |  |  |  |  |  |
| **Model 3** |  |  |  |  |  |  |  |  |
| As continuous (per SD) | 1.07 | 0.06 | 0.02 | 2.71 | 1.02 | 1.14 | 3013.3 | 0.006 |
| Interquartile |  |  |  |  |  |  |  |  |
| Quartile1 (1.52–296.47) | Ref. |  |  |  |  |  |  |  |
| Quartile2 (296.47–429.00) | 1.53 | 0.42 | 0.22 | 1.89 | 0.98 | 2.40 | 3000.1 | 0.059 |
| Quartile3 (429.00–620.00) | 1.82 | 0.59 | 0.21 | 2.78 | 1.20 | 2.82 |  | <0.001 |
| Quartile4 (620.00–28397.27) | 2.49 | 0.91 | 0.2 | 4.46 | 1.69 | 3.77 |  | <0.001 |
| Abbreviations: SII, systemic immune‐inflammation index. OR, odds ratio; CI, confidence interval; SE, standard error; AIC, Akaike Information Criterion. ^a^ The associations between SII levels and the risks of Parkinson's disease are presented as ORs (95% CI). Model 1 did not adjust for any covariates. Model 2 adjusted for age (years), gender, race, education level, marital status. Model 3 further adjusted for alcohol use, smoking—cigarette use, body mass index, sleep disorders, hypertension, diabetes, coronary heart disease and stroke based on Model 2. | | | | | | | | |
|  |  |  |  |  |  |  |  |  |
|  |  |  |  |  |  |  |  |  |
|  |  |  |  |  |  |  |  |  |
|  |  |  |  |  |  |  |  |  |
|  |  |  |  |  |  |  |  |  |
|  |  |  |  |  |  |  |  |  |
|  |  |  |  |  |  |  |  |  |

| Supplementary Table 3. Complete summary of RCS models with different knots. | | | | | | | |
| --- | --- | --- | --- | --- | --- | --- | --- |
| Knots | Effect | SE | 95%CI | | P for non-linearity | P overall | AIC |
|  |  |  | Lower | Upper |  |  |  |
| 4 | 0.77 | 0.12 | 0.53 | 1.01 | <0.001 | <0.001 | 3252.75 |
| 3 | 0.71 | 0.12 | 0.47 | 0.96 | <0.001 | <0.001 | 3255.78 |
| 5 | 0.68 | 0.18 | 0.32 | 1.04 | <0.001 | <0.001 | 3254.38 |
| Abbreviations: CI, confidence interval; SE, standard error; AIC, Akaike Information Criterion | | | | | | | |
|  |  |  |  |  |  |  |  |
|  |  |  |  |  |  |  |  |
